# Genistein-Inhibited Cancer Stem Cell-Like Properties and Reduced Chemoresistance of Gastric Cancer

**DOI:** 10.3390/ijms15033432

**Published:** 2014-02-25

**Authors:** Weifeng Huang, Chunpeng Wan, Qicong Luo, Zhengjie Huang, Qi Luo

**Affiliations:** 1 Department of Surgical Oncology, First Affiliated Hospital of Xiamen University & Xiamen Cancer Center, Xiamen 361003, Fujian, China; E-Mails: huangwf006@163.com (W.H.); qcluo@xmu.edu.cn (Q.L.); h74zj@126.com (Z.H.); 2 Medical College, China Three Gorges University, Yichang 443002, Hubei, China; 3 Jiangxi Key Laboratory for Postharvest Technology and Nondestructive Testing of Fruits & Vegetables, College of Agronomy, Jiangxi Agricultural University, Nanchang 330045, Jiangxi, China; E-Mail: lemonwan@126.com

**Keywords:** genistein, gastric cancer stem cell, chemoresistance

## Abstract

Genistein, the predominant isoflavone found in soy products, has exerted its anticarcinogenic effect in many different tumor types *in vitro* and *in vivo*. Accumulating evidence in recent years has strongly indicated the existence of cancer stem cells in gastric cancer. Here, we showed that low doses of genistein (15 μM), extracted from *Millettia nitida* Benth var hirsutissima Z Wei, inhibit tumor cell self-renewal in two types of gastric cancer cells by colony formation assay and tumor sphere formation assay. Treatment of gastric cancer cells with genistein reduced its chemoresistance to 5-Fu (fluorouracil) and ciplatin. Further results indicated that the reduced chemoresistance may be associated with the inhibition of ABCG2 expression and ERK 1/2 activity. Furthermore, genistein reduced tumor mass in the xenograft model. Together, genistein inhibited gastric cancer stem cell-like properties and reduced its chemoresistance. Our results provide a further rationale and experimental basis for using the genistein to improve treatment of patients with gastric cancer.

## 1. Introduction

Soy isoflavones have been identified as dietary components contributing to relatively lower rates of different types of cancer in Asian counties including China. *Millettia nitida Benth* var hirsutissima Z Wei (Fengcheng Jixueteng in Chinese) is a perennial herb distributed in Jiangxi and Fujian provinces of Southeast China [1]. In Chinese folk medicine, it is used to treat dysmenorrhea, irregular menstruation, rheumatic pain, aching pain, as well as paralysis [2]. Genistein (4′,5,7-trihydroxyisoflavone), the predominant isoflavone was found in soy products. Laboratory research from the last few decades have provided convincing evidence of the inhibitory effects of genistein on various cancer cells including breast, prostate, gastric, ovarian cancer cell [3]. It has been demonstrated that genistein functions as a promising chemopreventive agent to inhibit carcinogenesis through the modulation of genes that intimately related to the regulation of programmed cell death and cell cycle [4,5]. Additionally, genistein has been shown to inhibit to the angiogenesis and metastasis [6,7], imply the pleiotropic effects of genistein on the inhibition of carcinogenis and cancer cell growth. There may be other mechanisms of inhibition of cancer by genistein that are as yet undiscovered.

Unlike most cancer cells with a tumor, the cancer stem cells (CSCs) hypothesis suggests that they are a unique subpopulation in the tumors, which posses the ability to initiate tumor growth, self-renewal, and resist chemotherapeutic drugs, thereby causing relapse of the disease [8]. Thus, drugs that inhibited cancer cell self-renewal and reduced chemoresistance offer great promise for cancer treatment. Worldwide, gastric cancer is the fourth most common cancer and the second highest cause of cancer-related morbidity (1 million deaths per year) after lung cancer [9]. Increasing evidences have indicated the existence of gastric cancer stem cells (GCSCs). Shigeo TakaiShi *et al.* showed for the first time that CD44 appears to be the most useful marker for prospective purification of GCSCs [10]. In the subsequent study using CD44 and CD54, GCSCs were successfully isolated from the blood of gastric cancer patients [11]. In a recent study, the CD90 Marker was used [12]. The CD44 and CD24 combination has also been used for the isolation attempt [13]. In addition, stem cell markers such as OCT4, Sox2, Nanog have been recommended for identifying GCSCs [14]. Moreover, cells isolated from the gastric cancer cell lines using the tumor sphere culture technique exhibited characteristics of CSCs with high expression levels of stem cell marks with a mutipotent capacity of differentiation and enhanced tumorigenicity [15].

As mentioned above, a number of reports have demonstrated the inhibiting carcinogenesis by genistein through the modulation of multiple regulatory pathways in the mammary tumor model, including programmed cell death, cell cycle, angiogenesis and metastasis. However, the potential inhibitory of genistein on the gastric cancer cell stem-like properties is still unclear. In the present study, our results demonstrated that gastric cancer cells treated with genistein inhibited the gastric cancer cell stem-like properties, such as self-renewal ability, drug resistance and tumorigenicity, which are associated with the decreased expression of stemness-related genes and the drug resistance gene *ABCG2*.

## 2. Results

### 2.1. Identification of Genistein

The isolated compound was identified by a combination of NMR and mass spectral data and by comparison of these to published in the literature.

**Figure 1.**
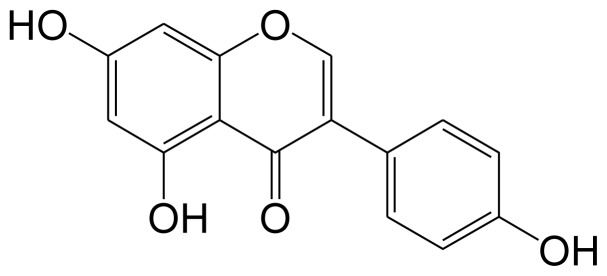
Chemical structure of the Genistein.

Compound **1**, colorless needle crystal (Figure 1); UV-vis (MeOH) λ_max_ = 269 nm; (−) ESI-MS, *m*/*z* 269.03 [M − H]^−^, calcd for molecular formula C_15_H_10_O_5_. ^1^H-NMR (600 MHz, CD_3_COCD_3_) δ: 13.02 (1H, s), 9.62 (1H, s), 8.52(1H, s), 8.16 (1H, s, H-2), 7.44 (2H, d, *J* = 8.5 Hz, H-2′, 6′), 6.88 (2H, d, *J* = 8.5 Hz, H-3′, 5′), 6.40 (1H, d, *J* = 2.0 Hz, H-6), 6.25 (1H, d, *J* = 2.0 Hz, H-8). ^13^C-NMR (150 MHz, CD_3_COCD_3_) δ: 154.2 (C-2), 124.0 (C-3), 170.1 (C-4), 163.7 (C-5), 99.8 (C-6), 164.8 (C-7), 94.2 (C-8), 159.1 (C-9), 106.0 (C-10), 123.0 (C-1′), 131.2(C-2′,6′), 115.8 (C-3′,5′), 158.4 (C-4′). The NMR data were consistent with the literature and compound 1 was identified as genistein [16].

### 2.2. Genistein Inhibited GCSCs Self-Renewal Properties and Negatively Correlated with GCSCs Characteristics

**Figure 2.**
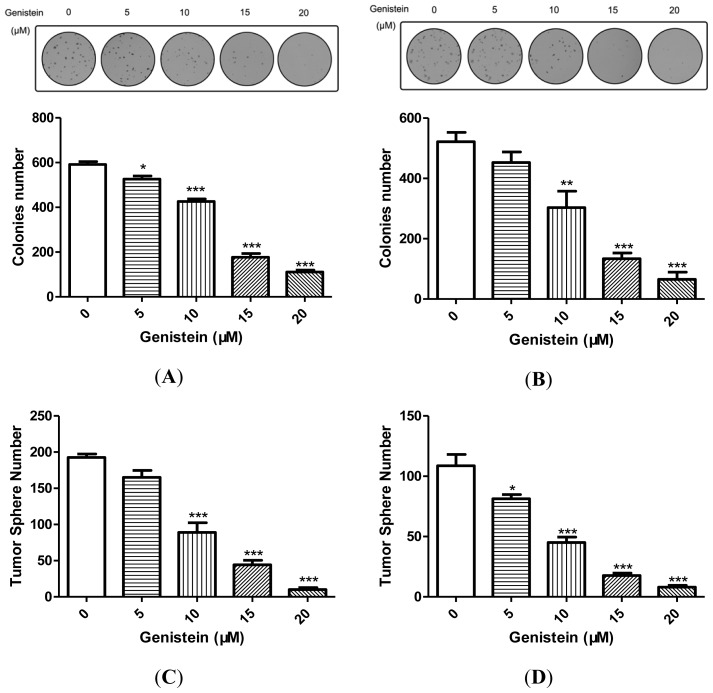

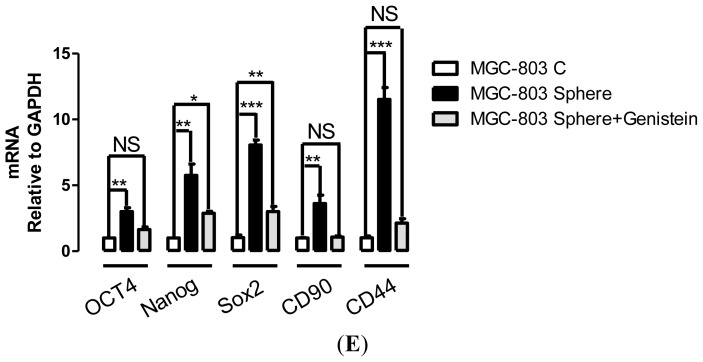
Genistein-inhibited gastric cancer stem cells (GCSCs) self-renewal properties and negatively correlated with GCSCs characteristics. (**A**) Genistein inhibited the colony formation capacity in MGC-803 in a dose-dependent manner. Representative photograph of colony was shown on the top. Statistical analysis was shown on the bottom; (**B**) Genistein inhibited the colony formation capacity in SGC-7901 in a dose-dependent manner. Representative photograph of colony was shown on the top. Statistical analysis was shown on the bottom; (**C**) Genistein inhibited the tumor sphere formation capacity in MGC-803 in a dose-dependent manner; (**D**) Genistein inhibited the tumor sphere formation capacity in SGC-7901 in a dose-dependent manner; (**E**) Several gastric stem cell markers expression in tumorspheres and monolayer cells. Data are the mean ± SEM of three independent experiments. * *p* < 0.05, ** *p* < 0.01, *** *p* < 0.001 *vs*. control. NS, no significance.

Tumor sphere assay and soft agar colony formation assay have been used to identity stem cell widely *in vitro* assays [17]. We first assayed the colony formation capacity in MGC-803 and SGC-7901 under the treatment of genistein. The results showed genistein inhibited the colony formation capacity in these cells in dose-dependent manner as shown in Figure 2A,B. Even 10 μM concentrations of genistein could cause significant inhibition of colony formation in MGC-803 (28.62%, *p* < 0.001, Figure 2A) and SGC-7901 (60.68%, *p* < 0.01, Figure 2B).

We then examined the tumor sphere formation capacity in MGC-803 and SGC-7901 under the treatment of genistein. Our results showed that the gastric cancer cells sphere formation capacity was inhibited by genistein in a dose-dependent manner, as shown in Figure 2. At 10 μM genistein, the inhibition efficiency of tumor sphere formation is 53.80% (MGC-803, *p* < 0.001, Figure 2C) and 58.58% (SGC-7901, *p* < 0.001, Figure 2D), respectively.

Moreover, we compared GCSCs marker expression in monolayer MGC-803 cells, MGC-803 spheres and MGC-803 spheres treated with 15 μM genistein by Real-Time PCR. The results showed that the spheres expressed much higher levels of GCSCs markers, such as *OCT-4*, *Sox2*, *Nanog*, *CD44*, and *CD90* than the monolayer cells. The induction of GCSCs markers were greatly suppressed in the spheres treated with 15 μM genistein compared with the spheres that were untreated (Figure 2E).

### 2.3. Genistein Reduced Gastric Cancer Cell Chemoresistance

**Figure 3.**
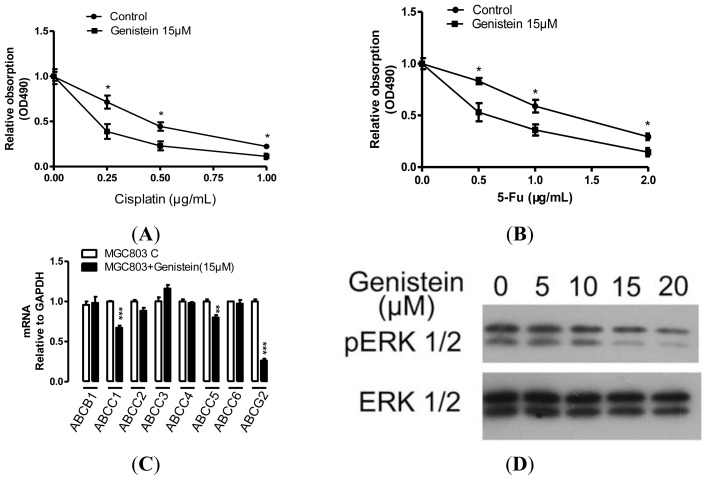
Genistein reduced gastric cancer cell chemoresistance. (**A**) Genistein reduced MGC-803 chemoresistance. MGC-803 pre-treated with genistein (15 μM) or not for 24 h, and then various concentration of cisplatin were used to treat MGC-803 cells. Cell viability was determined by MTT assay; (**B**) Genistein reduced MGC-803 chemoresistance. MGC-803 pre-treated with genistein (15 μM) or not for 24 h, and then various concentration of 5-Fu were used to treat MGC-803 cells. Cell viability was determined by MTT assay; (**C**) Drug resistant genes expression in MGC-803 under the treatment of genistein (15 μM); (**D**) Genistein inhibited ERK 1/2 activity. pERK 1/2 and ERK 1/2 expression in MGC-803 treated with indicated concentration of genistein were detected by western blotting. Data are the mean ± SEM of three independent experiments. * *p* < 0.05, ** *p* < 0.01, *** *p* < 0.001 *vs*. control.

Studies in the past have suggested that chemoresistance is another characteristic of CSCs [18]. We next investigated whether genistein would influence the chemoresistance of MGC-803 cells. For this purpose, MGC-803 pre-treated with genistein (15 μM) or not for 24 h and then various concentrations of two chemotherapy drugs, 5-Fu and cisplatin, were used to treat the cells. As showed in Figure 3A,B, genistein enhanced chemosensitivity to these chemotherapy drugs. Moreover, we detected chemoresistant genes expressed in MGC-803 under the treatment of genistein (15 μM). As shown in Figure 3C, ABCC1, ABCC5 and ABCG2 expression were repressed under the treatment of genistein (15 μM). Especially, the inhibition efficiency of ABCG2 was about 73.73% under the treatment of genistein in compared with the control.

It has been reported that ERK 1/2 activity plays an important role in regulating the ABCG2 expression. Genistein has been found to be effective in preventing cytokine-induced ERK 1/2 activation. To evaluate the role of ERK 1/2 activity in genistein reduced gastric cancer cell chemoresistance we detected the ERK 1/2 and phospho-ERK 1/2 in MGC-803 under the treatment of genistein. As shown in Figure 3D, phospho-ERK 1/2 expression was inhibited by genistein in a dose-dependent manner; at the same time, the total ERK 1/2 expression was unchanged.

### 2.4. Genistein Reduced the Tumorigenicity *in Vivo*

**Figure 4.**
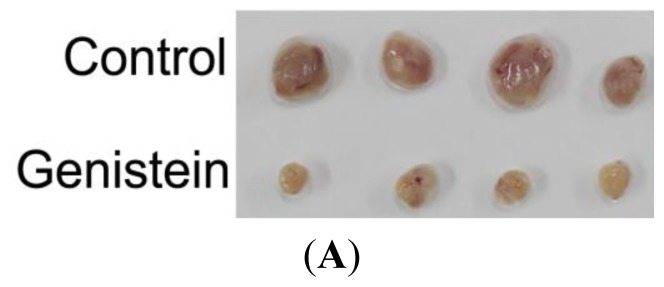

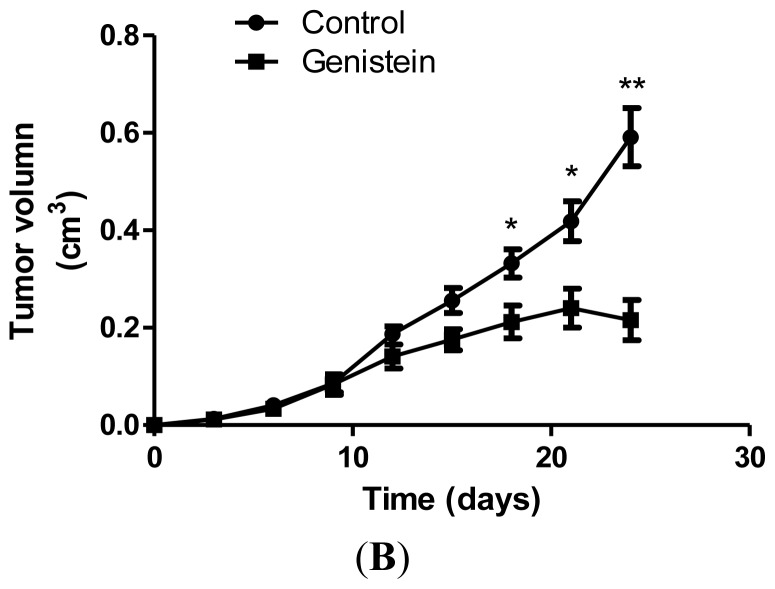
Genistein reduced the tumorigenicity *in vivo*. (**A**) Tumors produced by MGC-803. MGC-803 Cell (5 × 10^6^) were injected subcutaneously in the right flank of nude mice per mouse respectively (*n* = 4). And when the tumors developed in 7 days, the mice were was randomly distributed into two groups, and were untreated or treated by i.p. injections every day with genistein (1.5 mg/kg); (**B**) Tumor growth curves were monitored during the experimental period (*n* = 4) Data represent the mean ± SEM of three independent experiments. * *p* < 0.05, ** *p* < 0.01 *vs*. control.

It has been shown that the tumorigenicity *in vivo* correlates with the sphere formation ability of tumor cells *in vitro*. To test the effect of genistein on the tumorigenicity of gastric cancer cells, MGC-803 Cell (5 × 10^6^) were inoculated in to nude BALB/C mice. When the tumors had developed for 7 days, the mice were randomly distributed into two groups, and were untreated or treated with genistein. We found the size and weight of xenograft tumors treated with genistein was significantly smaller than the control tumors (Figure 4). These results thus demonstrated that genistein efficiently attenuated the tumorigenicity of gastric cancer cells.

## 3. Discussion

Genistein, the predominant component isoflavone in soy products, has been found to inhibit various cancer cells. It has been demonstrated that genistein prevents carcinogenesis by modulating multiple signaling pathways, such as programmed cell death, cell cycle, angiogenesis and so on [3]. Cancer stem cells hypothesis suggested that CSCs is the main cause of relapse in cancer patients [17]. We speculated that inhibiting CSCs properties may be a potential mechanism of preventing carcinogenesis by genistein. The tumor sphere assay has been used to identity stem cells, as shown in Figure 2C,D. CSCs markers were greatly induced in our experimental system. Our results demonstrated that genistein inhibits gastric cancer cell self-renewal capacity (Figure 2A–D). Consistent with the inhibitory effects, genistein suppressed the GCSCs markers induction (Figure 2E).

Chemoresistance is another characteristic of CSCs [17]. Our results showed that genistein enhanced gastric cancer cell chemosensitivity to 5-Fu and cisplatin. The chemosensitivity is associated with downregulation of ABCC1, ABCC5, ABCG2 and ERK 1/2 activity (Figure 3C,D). Genistein inhibited ABCG2 mRNA expression. At the same time, genistein inhibited the tumor sphere formation. Our results are consistent with other reports that ABCG2 not only plays a major role in multidrug resistance but can also be characterized as a CSCs marker [19].

ERK 1/2 have been demonstrated to play an important role in regulating the ABCG2 expression [20], and genistein could prevent ERK 1/2 activity [21]. We found that genistein inhibited phospho-ERK 1/2 in dose-dependent manner. Our data indicated that genistein enhanced gastric cancer cell chemosensitivity and is associated with the suppression of ERK 1/2 activity.

## 4. Experimental Section

### 4.1. Extraction and Isolation

The stems of the *Millettia nitida Benth* var hirsutissima Z Wei were collected locally from Fengcheng (Fengcheng, Jiangxi, China) in May 2008. Voucher specimens (ID: 200805) are deposited in the Medical College, China Three Gorges University (Yichang, Hubei, China). The stems (2 kg) were powered and extracted exhaustively with ethanol (3 × 8 L) at room temperature to yield a dried ethanol extract (178 g). The extract was re-suspended in H_2_O (3 L) and partitioned with CHCl_3_ (3 × 3 L), EtOAc and *n*-buthanol respectively. The CHCl_3_ extract (26.8 g) was chromatographed on a silica gel column (4 × 40 cm) eluting with a gradient system of Petroleum Ether/Acetone (10:1 to 1:2, *v*/*v*) to afford five sub-fractions (Fr.1–Fr.5) which were combined based on TLC analyses. Fraction Fr.4 was further separated by Sephadex LH-20 (Springup, Beijing, China) and semi-preparative HPLC (SHIMADZU, Kyoto, Japan) to yield compound **1** (30.2 mg).

### 4.2. Cell Cultures

Human gastric cancer cell lines, MGC-803 and SGC-7901, were purchased from Institute of Cell Biology (Shanghai, China, http://www.cellbank.org.cn). Cells were maintained in Royal Park Memorial Institute-1640 (RPMI-1640). All cell culture media were supplemented with 10% fetal bovine serum (FBS), and 1% of penicillin-streptomycin (all from Invitrogen, Carlsbad, CA, USA, http://www.invitrogen.com).

### 4.3. Soft Agar Colony Formation Assay

Triplicate samples of cells (1 × 10^3^) were resuspended in 1 mL of RPMI-1640 medium containing 0.3% low-melt agarose, 10% fetal bovine serum, 1% of penicillin-streptomycin. The cell mixture was plated on top of solidified layer with the same RPMI-1640 medium (Invitrogen, Carlsbad, CA, USA) contain 0.6% low-met agarose. Plates were incubated for 3 weeks at 37 °C in 5% CO_2_ in humidified incubator. MGC-803 and SGC-7901 cells treated with indicated concentration of genistein were plated in 0.3% agrose, and colonies were counted 3 weeks later. Then Colony formation was stained with 0.01% crystal violet and photographed and counted.

### 4.4. Tumorsphere Culture

Tumorspere cultures were cultured in ultralow attachment six-well plate (Corning, Lowell, MA, USA) in suspention (500 cells/mL) in serum-free DMEM/F12 media, supplemented with 20 ng/mL epidermal growth factor (FGF, Sigma-Aldrich, Shanghai, China), 4 μg/mL insulin (Sigma-Aldrich, Shanghai, China), B27 supplement (1×, Invitrogen, Carlsbad, CA, USA), 1% of penicillin-streptomycin in humidified incubator at 37 °C in 5% CO_2_. Tumor sphere formation was tested by placing gastric cancer cells in presence or absence of genistein under the conditions mentioned above. After 7 day incubation, the solid spheres were photographed and counted. The sphere size varies greatly from less than 50 μM to around 250 μM. With tumorspheres, the cells appear fused together and it is difficult to distinguish them as individual cells. With aggregated cells that were not counted, you can still see individual cells attached to one another.

### 4.5. RNA Extraction, RT-PCR and Quantitative Real-Time PCR

Total RNA was extracted using TRIZOL Reagent (Invitrogen, Carlsbad, CA, USA) and reverse transcribed with R-PCR Quick Master Mix (Toyoba, Dalian, China) to produce cDNA. The primer sequences listed in Table 1 were used for Quantitative Real-Time PCR. Real-Time PCR was performed using SYBR Green-based detection in LightCycler^®^480 (Roche, Indianapolis, IN, USA) according to the manufacture’s instructions. *OCT-4*, *Sox2*, *Nanog*, *CD44*, and *CD90* expression in monolayer MGC-803 cells, MGC-803 spheres and MGC-803 spheres treated with 15 μM genistein were detected by Real-Time PCR. ABCB1, ABCC1, ABCC2, ABCC3, ABCC4, ABCC5, ABCC6 and ABCG2 expression in MGC-803 cells treated with 15 μM genistein were detected by Real-Time PCR. GAPDH (glyceraldehyde-3′phosphate dehydrogenase) levels were used as normalization controls.

#### 4.5.1. Chemoresistance Assay

The MTT assay (Cell titer 96^®^ Aqueous One Solution Cell Proliferation Assay, Promega, Beijing, China) was used to assess the rates of resistance to drugs. Briefly, gastric cancer cells (2 × 10^3^/well) were seeded in 96-well plates. After 24 h in presence or absence of genistein, the indicated concentration of chemotherapeutic drugs, 5-Fu (Sigma-Aldrich, Shanghai, China) and cisplatin (Sigma-Aldrich, Shanghai, China) were treated. 72 h later, the MTT assay was performed using iMarkmicroplate Absorbance Reader (Bio-RAD, Richmond, CA, USA) according to the manufacturer’s instructions.

### 4.6. Cell Extraction and Western Blotting

Western Blots were performed according to the protocols described previously [22]. The Immobilon Western Chemiluminescent HRP Substrate Kit (Millipore, MA, USA) was used to detect the results. Primary Antibody are ERK1/2 (AM076, 1:500; Beyotime, Nanjing, China), and phospho-ERK1/2 (AM071, 1:500; Beyotime, Nanjing, China).

### 4.7. Tumor Growth in Xenografts

MGC-803 Cell (5 × 10^6^) were injected subcutaneously in the right flank of 8 female 6-week-old nude mice (Laboratory Animal Center, Xiamen University, Xiamen, China) per mouse respectively. When the tumors had developed for 7 days, the mice were randomly distributed into two groups, and were untreated or treated by i.p. injections every day with genistein (1.5 mg/kg). Tumor volumes (*T*v) were measured every 3 days and calculated with the formula: *T*v = *L* (Length) × *W*^2^ (Width)/2.

### 4.8. Statistical Analysis

Results are expressed as the means ± SEM. Statistical significance was determined by Student’s *t* test or a one-way or two-way analysis of variance (ANOVA) followed by Turkey’s test, as appropriate using Graphpad Prism statistics software (Graphpad Software, CA, USA). A *p*-value < 0.05 was considered statistically significant (* *p* < 0.05, ** *p* < 0.01, *** *p* < 0.001).

## 5. Conclusions

Our present results showed that genistein inhibited GCSCs properties *in vitro*, reduced the gastric cancer cell tumorigenicity *in vivo*, enhanced chemosensitivity of gastric cancer cells and provided an experimental basis for using the genistein to improve treatment of patients with gastric cancer.

## Figures and Tables

**Table 1.** Primers used for quantitative real-time PCR.

Gene	Primer direction	Sequences
*OCT*4	Forward	GGTATTCAGCCAAACGACCAT
	Reverse	ACGAGGGTTTCTGCTTTGCA
*Nanog*	Forward	GGTTCCAGAACCAGAGAATGAAA
	Reverse	GTTGCTCCACATTGGAAGGTT
*SOX*2	Forward	CTCGCCCACCTACAGCAT
	Reverse	GACTTGACCACCGAACCC
*CD*90	Forward	AGGGTTGGAGAAGGAGGTA
	Reverse	CTGACTTGGAGGCTGTGG
*CD*44	Forward	CTCATGGATCTGAATCAGATGGA
	Reverse	ACTGCAATGCAAACTGCAAGA
*ABCB*1	Forward	AGCTCGTGCCCTTGTTAGACA
	Reverse	GTCCAGGGCTTCTTGGACAA
*ABCC*1	Forward	GAGAGATCATCATCGATGGCAT
	Reverse	AGGGACGTCCAGACTTCTTCAT
*ABCC*2	Forward	GCTGCCGGTGGTCAGATTAT
	Reverse	AAGGCCTTCCAAATCTCCTCA
*ABCC*3	Forward	CGCACACCGGCTTAACACTAT
	Reverse	TCTCTGGCCATCCCGTAGAA
*ABCC*4	Forward	TGCCCTCACTGAAACAGCAA
	Reverse	GGATTCACAGTGCTGTCTCGAA
*ABCC*5	Forward	CAAGAGACCATCCGAGAAGCA
	Reverse	GGGAACTGTCGTTGGACAGAA
*ABCC*6	Forward	CAGTGCACTGTGCTGCTCATT
	Reverse	TGACTCCTGGGCCAGTCTGT
*ABCG*2	Forward	GGACTCAATGCAACAGGAAACA
	Reverse	TCAGGTAGGCAATTGTGAGGAA
*GAPDH*	Forward	TCTCCTCTGACTTCAACAGCGA
	Reverse	GTCCACCACCCTGTTGCTGT
